# A High-Temperature and Wide-Permittivity Range Measurement System Based on Ridge Waveguide

**DOI:** 10.3390/s25020541

**Published:** 2025-01-18

**Authors:** Rui Xiong, Yuanhang Hu, Anqi Xia, Kama Huang, Liping Yan, Qian Chen

**Affiliations:** School of Electronics and Information Engineering, Sichuan University, Chengdu 610064, China; xiongrui1@stu.scu.edu.cn (R.X.); xiaanqi@stu.scu.edu.cn (A.X.);

**Keywords:** ridge waveguide, complex relative permittivity, artificial neural network, scattering parameters, microwave measurement

## Abstract

Potential applications of microwave energy, a developed form of clean energy, are diverse and extensive. To expand the applications of microwave heating in the metallurgical field, it is essential to obtain the permittivity of ores throughout the heating process. This paper presents the design of a 2.45 GHz ridge waveguide apparatus based on the transmission/reflection method to measure permittivity, which constitutes a system capable of measuring the complex relative permittivity of the material under test with a wide temperature range from room temperature up to 1100 °C. The experimental results indicate that the system is capable of performing rapid measurements during the heating process. Furthermore, the system is capable of accurately measuring dielectric properties when the real part of the permittivity and the loss tangent vary widely. This measurement system is suitable for high-temperature dielectric property measurements and has potential applications in microwave-assisted metallurgy.

## 1. Introduction

In recent years, there has been a notable increase in the applications of microwave technology for various purposes. The reasonable applications of microwave technology will lead to great improvements in industry, a reduction in energy consumption, and the conservation of resources, thereby promoting sustainable development. In metallurgy, material properties are typically subjected to heating and drying processes. Traditional heating methods result in the majority of the heating energy being consumed by the equipment itself and the surrounding environment, which leads to obvious energy inefficiency. The energy consumption of conventional industrial drying represents over 15% of the total global energy consumed each year [1]. It is possible to direct the application of microwaves so that the energy is concentrated in the material to be treated [2]. Microwave heating represents an important method that is efficient, energy-saving, and easily automated [3]. It can be applied to drying treatments, sintering, and carbothermal reduction. In comparison with the traditional techniques of drying by radiation, microwave drying technology exhibits a number of advantages, including a high heating speed, high drying efficiency, easy temperature control, minimal energy consumption, and so forth. Furthermore, it can also be more effective in the protection of items during the drying process [4]. Singh et al. designed and developed a microwave sintering device that achieved product performance comparable to those of conventional sintering, and the process is also suitable for large-scale production in industrial applications [5]. Moreover, carbothermal reduction represents a pivotal process in metallurgical engineering. By accelerating the carbothermal reduction rate, the purity and overall quality of the resulting metallurgical engineering products can be enhanced. The integration of microwave reduction technology within the smelting process of carbon-containing minerals can expedite the heating rate of these minerals, thereby augmenting their value in metallurgical engineering [6].

However, the current application of this technology in the metallurgy field is not yet optimal. Several issues require further attention, one of which is the complex dielectric properties of materials being heated. In order to enhance the effectiveness of microwave utilization, it is imperative to obtain precise, complex relative permittivity data for the materials in question. The complex relative permittivity of a substance describes the interaction between electromagnetic waves and the medium [7,8,9].

A number of established techniques are currently employed for the measurement of permittivity. These include the resonant cavity method, free-space method, open-ended coaxial line method, and transmission line method [10,11,12,13,14,15,16,17,18,19,20]. Resonance methods are techniques for measuring the permittivity of materials, whereby a sample is inserted into a resonant cavity. Changes in the resonance frequency and quality factor (Q-factor) of the cavity are employed to calculate the complex relative permittivity of the material [21]. These methods offer high accuracy and sensitivity at a single frequency and are typically employed for low-loss and small-sized material measurements.

Free-space methods situate materials between two antennas in an open environment. Electromagnetic waves emitted by antennas interact with materials, resulting in reflection and transmission phenomena that can be quantified to ascertain the complex relative permittivity of materials. Free-space methods are versatile techniques with extensive applications in high-temperature permittivity measurements and remote sensing [13]. These methods necessitate larger sample sizes, and system calibration can prove challenging.

Open-termination coaxial line methods place a sample at the end of a transmission line, effectively creating an open termination. Reflection and transmission occur due to the interaction with the material as high-frequency signals pass through a transmission line and reach a terminated end. Reflection coefficients are measured and combined with transmission line theory to calculate the complex relative permittivity of the materials [22]. These methods are commonly used to measure the dielectric properties of liquids or semisolids.

In transmission/reflection methods, materials are treated as a two-port network for testing purposes by placement in a transmission line, such as a coaxial, waveguide, or microstrip line [23,24]. These methods offer the benefits of straightforward operation, precise measurement, and the potential for broadband assessments. They are frequently employed for the measurement of permittivity at high temperatures.

The transmission/reflection methodology for measurement of the permittivity of dielectrics was originally proposed by Nicolson et al. [24]. In these methods, the material under test is positioned on the transmission line, resulting in phenomena such as reflection, absorption, and transmission of electromagnetic waves. By establishing a relationship between scattering parameters and material permittivity, the complex relative permittivity can be determined by measuring these parameters on the transmission line [25].

In the metallurgical industry, heating ores is a common practice. The temperature during heating represents a pivotal parameter that exerts a profound influence on the permittivity of a given material. By undertaking a comprehensive investigation into the impact of this parameter on the dielectric properties of materials and identifying the relevant governing principles, it becomes possible to optimize the utilization of microwave energy for the processing and treatment of target materials. It is, therefore, imperative to accurately determine the complex relative permittivity of materials at varying temperatures.

It has been demonstrated that the permittivity of ores exhibits a significant increase in both the real and loss tangent values when subjected to a temperature of 1000 °C, resulting in enhanced conductivity and microwave losses [26]. At 2.45 GHz, the frequency commonly utilized for microwave heating in industry, the real part of the permittivity of certain ores has been observed to reach 110, while the loss tangent may reach 1, following a temperature increase to 1000 °C [27].

Conventional measurement devices operating at 2.45 GHz have a measurement range for the real part of permittivity from 1 to 80 [28]. Katali employed open-ended coaxial probes to ascertain the complex relative permittivity of assorted fruits and vegetables at 2.45 GHz [29], with the maximum recorded real part of permittivity reaching 80, which is close to the permittivity of water. Currently, most equipment designed to measure a broad range of complex relative permittivity is not suitable for use with the frequency bands typically employed for microwave heating in industrial contexts [30,31,32,33]. Erhu Ni utilized an extra-cavity evanescent waveguide method to measure the complex relative permittivity of materials with a high real part of permittivity [34]. By measuring frequency shifts and cavity Q-factors, it is possible to accurately calculate the complex relative permittivity for materials with a high real part and a low-loss tangent within the X-Band frequency. Time-domain spectroscopy can analyze the real part and loss tangent of high-permittivity materials (such as calcium stearate, alumina, zirconium titanate, and tin titanate) in the terahertz frequency range [35], where permittivity values range from 6 to 90. In order to ensure the accuracy of measurements made on materials exhibiting a wide range of fluctuations in both the real part of permittivity and loss tangent, the development of measurement devices capable of achieving precise results is of paramount importance.

Conventional permittivity measurements are typically undertaken utilizing analytical methods, with the resonant cavity perturbation serving as the classical theoretical basis for microwave measurement techniques [36,37]. Alternatively, numerical fitting techniques such as the Debye equation are employed [38]. However, these traditional approaches frequently present several limitations. These include the necessity for a more complicated modeling process, the multi-valued nature of the solution of the transcendental equations, and the stringent requirements on the size of the sample to be measured. To address these challenges, optimization algorithms were found to be suitable for data processing, including genetic algorithms and neural networks [39,40]. Neural networks are able to learn complex nonlinear mapping relationships through training, so they are more advantageous in dealing with high-dimensional data and achieving predictions. A neural network is a computational model that has the capacity to learn and discover potential patterns from large datasets, thus helping to predict or solve various problems and is also applicable to the measurement of permittivity [41,42,43].

Based on the preceding analysis, a ridge waveguide device has been designed to measure the complex relative permittivity of minerals at high temperatures accurately This device employs the transmission/reflection method, particularly suited to the wide range of permittivity variation observed in such materials. The device is connected to a vector network analyzer, which is used to measure the scattering parameters of the test material at 2.45 GHz and a range of temperatures from room temperature to 1100 °C. A neural network is then applied to determine the complex relative permittivity of the material from the measured scattering parameters.

## 2. Materials and Methods

### 2.1. Ridge Waveguide Measurement System

**Figure 1:**
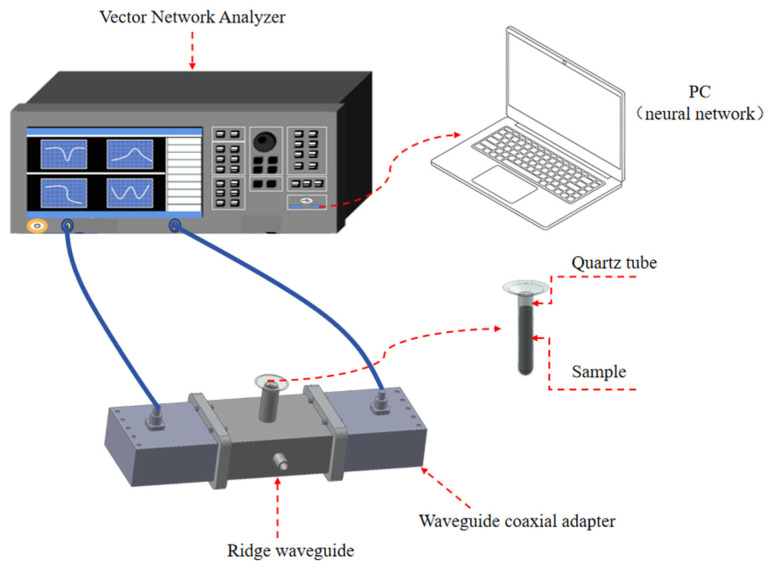
Schematic diagram of the complex relative permittivity measurement system.

At the present time, the commonly used microwave frequency in industrial microwave applications is 2.45 GHz, which serves as the basis for the design of this system. The schematic diagram of the measurement system is shown in Figure 1. The system is mainly composed of a ridge waveguide apparatus, two waveguide coax adapters, a vector network analyzer, coaxial cables, a quartz tube, and a PC.

**Figure 2:**
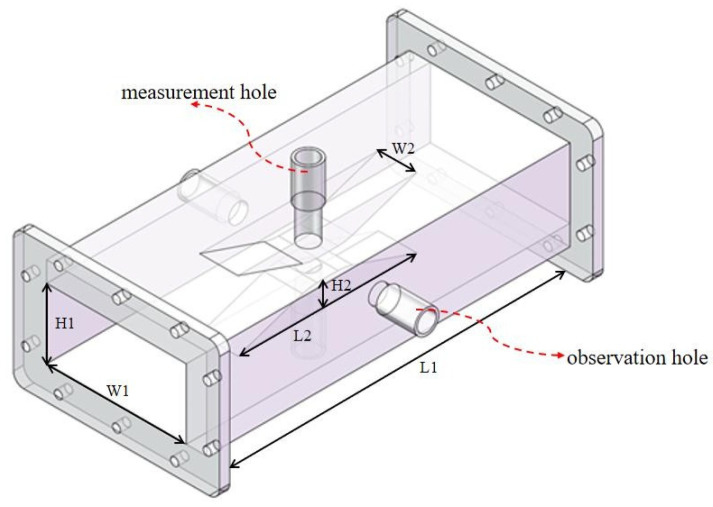
Schematic of the ridge waveguide.

The core device of the permittivity measurement system is a ridge waveguide based on the standard rectangular waveguide BJ26 (WR340), as illustrated in Figure 2. The apparatus comprises four principal parts: a rectangular waveguide, two ridges, two measurement holes, and two observation holes. The dimensions of the ridge waveguide are as follows: length (L1) 237 mm, width (W1) 86.36 mm, height (H1) 43.18 mm, ridge length (L2) 80 mm, ridge width (W2) 26 mm, and ridge height (H2) 17 mm.

**Figure 3:**
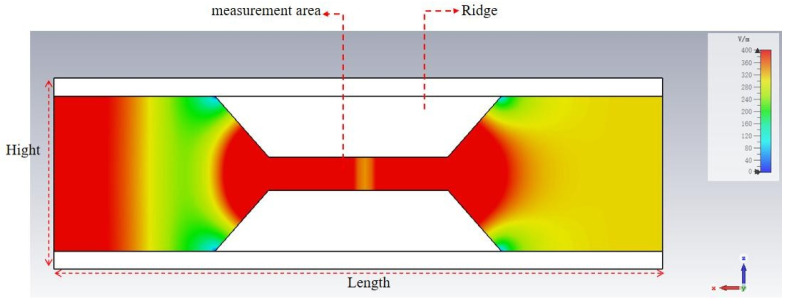
Simulated electric field on the longitudinal section of the ridge waveguide.

Adding two ridges in the middle of the broad wall, one at the top and the other at the bottom, allows the electromagnetic field to be focused between the two ridges. This structure enables the sample to be situated within the region of a strong electric field, thereby enhancing measurement sensitivity and reducing measurement uncertainty. Figure 3 illustrates the electric field within the ridge waveguide structure. The simulation results indicate that the ridge waveguide has the capacity to concentrate the electric field between two ridges. The measurement hole is situated in the center of the ridge and is used to position the material under examination. This aperture extends outward to form a circular waveguide, which operates in a cutoff state to avoid the leakage of electromagnetic energy. The observation hole is situated at the center of the narrow wall of the ridge waveguide, where the sample to be measured can be observed in real time. The observation holes extend outward to form a circular waveguide operating in the cutoff state.

### 2.2. Simulation Parameter Setting and Result Analysis

The complex relative permittivity is a useful parameter for evaluating the ability of a medium to absorb and reflect microwaves. It is represented by a real part (ε_r_′) and an imaginary part (ε_r_″), which are denoted as follows:

(1) $$\varepsilon_{r}={\varepsilon_{r}}^{\prime }-j{\varepsilon_{r}}^{\prime \prime }$$

The loss tangent (tanδ) is the ratio of the imaginary part to the real part of the complex relative permittivity. It represents the ability of a material to convert electromagnetic energy into thermal energy in a given state. Its expression is given by the following:

(2) $$\tan\delta =\frac{{\varepsilon_{r}}^{\prime \prime }}{{\varepsilon_{r}}^{\prime }}$$

**Figure 4:**
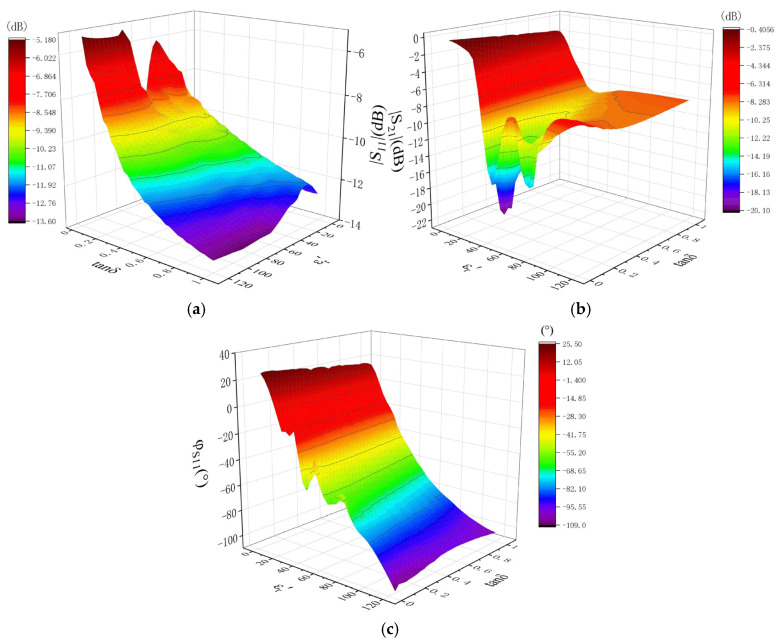
Scattering parameters simulations with changing complex relative permittivity. (**a**) |S_11_|—simulations with changing complex relative permittivity; (**b**) |S_21_|—simulations with changing complex relative permittivity; (**c**) φ_S11_—simulations with changing complex relative permittivity.

When the real part of the permittivity of the material to be measured varies from 1 to 120, and the loss tangent varies from 0.05 to 1, with a real part step size of 0.1 from 1 to 79.9 and 1 from 80 to 120, and a loss tangent step size of 0.05, the scattering parameters (|S_11_|, |S_21_| and φ_S11_) are simulated using full-wave simulation. The total number of samples for the simulation is 16,620. The simulation results are shown in Figure 4. The results demonstrate a notable variation in the scattering parameter with changes in the permittivity within the range of the real part from 1 to 120 and the loss tangent from 0.05 to 1. It can be observed that there is a linear relationship between the scattering parameter and the complex relative permittivity in most regions. These results provide a basis for accurately reconstructing the permittivity of a material using the scattering parameters. Additionally, the three parameters |S_11_|, |S_21_|, and φ_s11_ can be employed to reconstruct the permittivity of the material, thus avoiding the potential issue of multiple values that may occur when a single scattering parameter is used to calculate the complex relative permittivity. Furthermore, the reconstruction accuracy is enhanced.

### 2.3. Artificial Neural Network (ANN)

In this paper, an artificial neural network algorithm is used to reconstruct the complex relative permittivity of the material by measuring the scattering parameters [29]. A neural network is an artificial intelligence computational model that is utilized to simulate the structural organization of neural networks within the human brain. It consists of multiple layers of neurons and the weights connecting them. Each neural layer receives an input, processes it through weighted summation and activation functions, and produces an output. The neural network learns the complex nonlinear relationship between input and output by dynamically adjusting the weights of each neural layer. The parameters of the neural network are optimized by minimizing the discrepancy between the predicted and actual outputs, thereby enhancing accuracy and generalizability.

**Figure 5:**
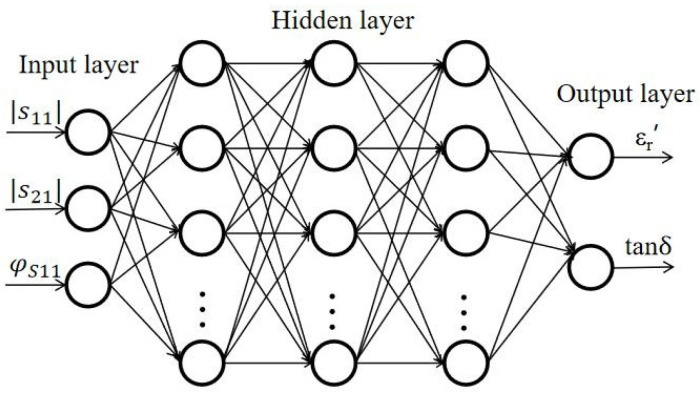
Schematic diagram of the neural network model.

The neural network structure for the complex relative permittivity reconstruction from scattering parameters is shown in Figure 5. The input vectors of the input layer are the scattering parameters (|S_11_|, |S_21_|, and φ_s11_), and the output layer outputs two dielectric characteristic vectors (ε_r_′ and tanδ). In this paper, a multi-layer fully connected neural network is employed. The introduction of nonlinearity through an activation function in each hidden layer serves to enhance the expressive and predictive performance of the model. For example, ReLU is one of the commonly used activation functions [44]. In the data preprocessing stage, the input features are normalized. This helps scale the data to a similar range, avoiding the influence of differences between different feature values on model training. The model iterates through training on the training set and uses a backpropagation algorithm to adjust the parameters of the model dynamically to minimize the loss function [45]. The established neural network model is then applied to predict new samples.

**Figure 6:**
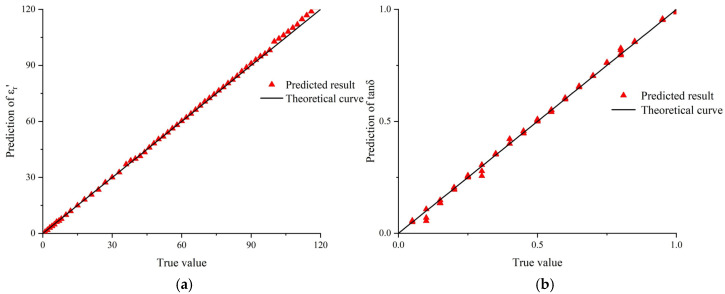
Permittivity prediction results. (**a**) Prediction effectiveness of the real part of permittivity; (**b**) prediction effectiveness of the loss tangent.

In order to validate the prediction performance of the neural network, 60 sets of sample data not used for training are taken from the simulated data as input. Subsequently, the trained neural network model is employed to obtain the output, which is illustrated in Figure 6. The closer the relationship between the predicted values and the actual permittivity values is to the theoretical curve, the higher the accuracy of the neural network’s predictions. The mean square errors of the predicted real part and loss tangent of the permittivity are 3.34% and 5.87%, respectively, thereby substantiating the credibility of the neural network model.

## 3. Results and Discussion

**Figure 7:**
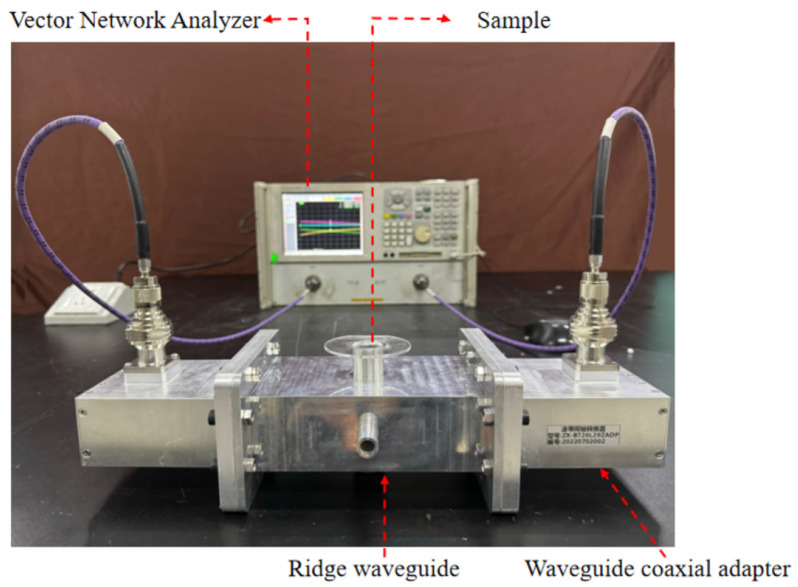
Photo view of the permittivity measurement system.

The reliability and accuracy of the measurement system were verified at room temperature. The photograph of the permittivity measurement system is shown in Figure 7. The fundamental apparatus employed in the measurement process, the ridge waveguide, was connected at both ends to a vector network analyzer via waveguide-to-coax adapters and coaxial cables. During the experiment, the scattering parameters were obtained by sampling with a test tube, which was inserted into the measurement hole of the waveguide for measurement. In practice, the measurement process is related to the state of the sample. Following the act of sampling, both liquid and solid powders can be measured directly with a test tube. Certain solids can be cut into cylindrical shapes and placed into the test tube. Alternatively, solids can be made into powder and compacted into cylindrical shapes using a mold before being placed into the test tube and measured. The sample is required to fill a cylindrical area with a diameter of 15.5 mm and a height of 45 mm, resulting in a volume of approximately 8.5 mL. Subsequently, the measured scattering parameters are entered into a PC, whereupon the complex relative permittivity of the sample is reconstructed through the application of artificial neural network algorithms.

**Figure 8:**
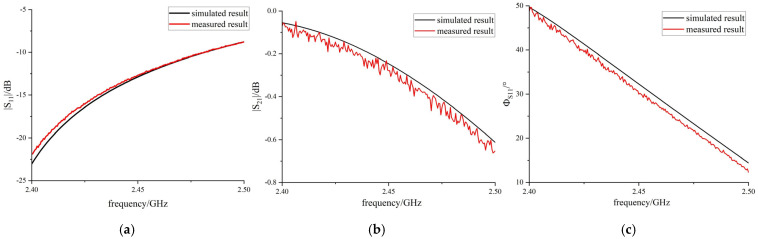
Comparison between simulation and measurement for air. (**a**) Comparison between simulated and measured |S_11_|; (**b**) comparison between simulated and measured |S_21_|; (**c**) comparison between simulated and measured φ_S11_.

In this paper, the measured scattering parameters are employed to reconstruct the permittivity, and a comparison is presented between the measured and simulated data, as shown in Figure 8. It is observed that there is consistency between the measured and simulated scattering parameters at 2.45 GHz when the measured material is air. The agreement between the measured and the simulated results provides a foundation for accurately measuring the complex relative permittivity in the experiments.

Ethanol is a widely used chemical reagent; therefore, we chose ethanol, DI water solutions, and their mixtures for measurement at 2.45 GHz frequency. The real part of the permittivity and the loss tangent values of these solutions were measured at a frequency of 2.45 GHz using the ridge waveguide system and the Keysight N1501A dielectric probe kit, respectively. To eliminate random factors and improve the reliability of the experiment, each sample was measured three times, and the average of the three measurements was taken. The results of the experiments are shown in Table 1.

It can be seen from the measurement results in Table 1 that the ridge waveguide measurement system is capable of accurately measuring the complex relative permittivity of ethanol, DI water solutions, and their mixtures at 2.45 GHz.

The relative error of the real part of the complex relative permittivity measurement is within 6.6%, and the relative error of the loss tangent measurement is also within 10.3%. The reagent with the largest measurement error in the experiment was a solution mixed in a ratio of 9:1. The source of the error may be attributed to the fact that the access to the cables, N-SMA adapters, and waveguide coax adapters between the vector network analyzer and the measurement apparatus introduces losses, which have an impact on the accuracy of the measurement. Furthermore, the accuracy of measurement results can be influenced by factors such as errors associated with measurement instruments, deviations in dimensional specifications during machining, and errors in reconstruction.

To confirm the capability of the system to measure a broad range of complex relative permittivity, measurements were conducted on high-dielectric ceramic samples, with a relative permittivity of 108 and loss tangents of 0.275. The main components of the ceramic samples are TiO_2_, SiO_2_, MgO, and a few rare-earth elements [46,47,48,49,50].

The test results are shown in Table 2. The results indicate that the ridge waveguide system can measure materials with a real part of permittivity greater than 100, providing a reliable basis for high-temperature testing of ores.

**Figure 9:**
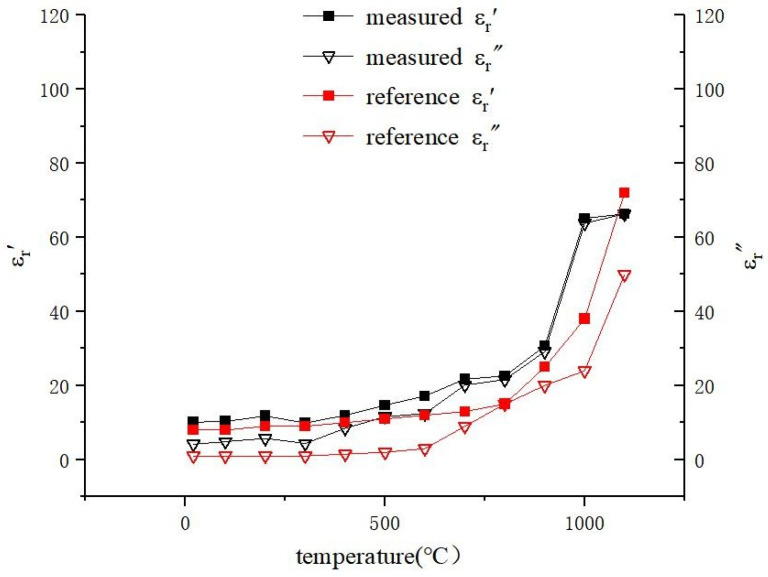
Temperature dependence of the complex relative permittivity of ore.

A series of experiments were conducted with the objective of quantifying the permittivity of the ore in response to a temperature increase. The materials utilized were hematite ore, exhibiting an iron grade of approximately 64%. The primary composition of the material was identified as hematite (Fe_2_O_3_), with a minor component of goethite (FeO(OH)) and silicon dioxide (SiO_2_). The hematite samples were subjected to a grinding process, resulting in a granular form. The samples were then analyzed using a laser particle size analyzer, which yielded an average particle size of 106 µm. Subsequent to this, the granular material was compressed into a cylindrical shape under a pressure of 6 MPa for a duration of 30 s. The density of the pressed sample was 3.368 g/cm^3^. To ensure that the samples were maintained in an inert gas atmosphere and prevented from reacting with oxygen at increasing temperatures, argon was introduced into the test tube to expel oxygen. Subsequently, the scattering parameters were measured by placing the test tube in the waveguide test hole at room temperature. Thereafter, the test tube was subjected to heating in a muffle furnace. The temperature within the muffle furnace was elevated from room temperature to 1100 °C, with the scattering parameters being recorded at 100 °C intervals. The resulting data were input into a neural network to determine the complex relative permittivity of hematite at different temperatures. The results are shown in Figure 9.

As illustrated in Figure 9, the alteration in the complex relative permittivity of the sample from room temperature to 1100 °C can be distinguished into two distinct phases. In the initial stage, which spans the temperature range from room temperature to 800 °C, the complex relative permittivity is relatively stable. The second stage is from 900 °C to 1100 °C, during which the complex relative permittivity increases markedly as a result of the shrinkage and sintering of the sample at high temperatures. The trend of temperature dependence of the complex relative permittivity of ore is almost consistent with reference [29]. The observed difference may be attributable to variations in sample composition and preparation processes, thereby validating the accuracy of the measurements obtained using the ridge waveguide system.

## 4. Conclusions

This paper presents a ridge waveguide apparatus-based permittivity measurement system that is capable of accurately reconstructing the complex relative permittivity of the material to be measured rapidly. This is achieved through the utilization of the transmission/reflection method, combined with full-wave simulation and neural network algorithms. The real part and loss tangent of the complex relative permittivity of ethanol, DI water solutions, and their mixtures at room temperature were measured using the system, and the measurements were found to be in good agreement with the Keysight commercial probe, thus verifying the accuracy of the system’s measurements.

Furthermore, the system was employed with a heating device to achieve rapid measurements of complex relative permittivity from room temperature to 1100 °C at 2.45 GHz. The measured complex relative permittivity of hematite with increasing temperature and at high temperatures is consistent with the literature, indicating that the measurement system is capable of achieving accurate measurements over a wide dynamic range of 1–120 in the real part of the permittivity and 0.05–1 in the loss tangent. The system can assist the microwave metallurgy industry in measuring ores under high-temperature conditions, with a wide range of variations in complex relative permittivity. In addition, the system is straightforward to operate, sample preparation is uncomplicated, the sample dosage is minimal, and there are potential applications in microwave energy industrial contexts.

## Figures and Tables

**Table 1:** The permittivity of solutions at 2.45 GHz.

Liquid to Be Measured	Waveguide (ε′)	Probe (ε′)	Errors (%)	Waveguide (tanδ)	Probe (tanδ)	Errors (%)
Water/ethanol = 0:10	7.642	7.940	3.753	0.883	0.894	1.230
Water/ethanol = 1:9	13.968	13.100	6.626	0.846	0.806	4.963
Water/ethanol = 2:8	20.706	20.620	0.417	0.667	0.644	3.571
Water/ethanol = 3:7	27.779	28.505	2.547	0.528	0.525	0.571
Water/ethanol = 4:6	34.539	35.293	2.136	0.468	0.453	3.311
Water/ethanol = 5:5	41.734	43.084	3.133	0.405	0.378	7.143
Water/ethanol = 6:4	48.361	51.120	5.397	0.352	0.328	7.317
Water/ethanol = 7:3	56.774	57.325	0.961	0.259	0.270	4.074
Water/ethanol = 8:2	63.052	65.822	4.208	0.219	0.210	4.286
Water/ethanol = 9:1	67.147	71.578	6.190	0.171	0.155	10.322
Water/ethanol = 10:0	75.593	77.370	2.297	0.122	0.123	0.813

**Table 2:** The permittivity of ceramic material at 2.45 GHz.

Reference Value (ε′)	Test Value (ε′)	Reference Value (tanδ)	Test Value (tanδ)
105	108	0.275	0.286

## Data Availability

The data that supported the findings of this study are available from the corresponding author upon reasonable request.
